# The Efficacy of Trigger Site Surgery in the Elimination of Chronic Migraine Headache: An Update in the Rate of Success and Failure

**DOI:** 10.7759/cureus.54504

**Published:** 2024-02-20

**Authors:** Eyad Faizo, Ahmad Fallata, Iman Mirza, Ahmed K Koshak, Yasmeen T Bucklain, Reema Alharbi, Abdulrahman Tasji, Taha Tasji, Ahmed Kabbarah

**Affiliations:** 1 Department of Surgery, University of Tabuk, Tabuk, SAU; 2 Department of Internal Medicine, University of Tabuk, Tabuk, SAU; 3 Department of Family and Community Medicine, University of Tabuk, Tabuk, SAU; 4 Faculty of Medicine, Fakeeh College for Medical Sciences, Jeddah, SAU; 5 Department of Medicine, Fakeeh College for Medical Sciences, Jeddah, SAU

**Keywords:** prognosis, decompression, surgery, trigger sites, migraine headache

## Abstract

Migraine headache (MH) is a prevalent neurovascular disorder that affects approximately 15% of the global population. They are more common in women and typically affect young and middle-aged individuals. Chronic MH is characterized by headaches occurring on ≥15 days per month for over three months. While only 5% of MHs are refractory, about 20%-50% do not respond to pharmacologic treatments. As a result, surgical interventions have emerged as an alternative method to eliminate MH since 2000 AD. These surgical treatments primarily target the peripheral mechanisms of MH, focusing on common trigger sites. Migraine surgery involves neurolysis of sensory branches of trigeminal and occipital nerves that supply the face and back of the head. Numerous clinical studies conducted between 2000 and 2021 have extensively described surgical interventions and their prognostic outcomes. After surgery, up to 80% of patients reported complete elimination of headaches, while 20%-35% experienced no relief. The failure to achieve complete elimination of MH can be attributed to various factors. The most common reason for a partial clinical response is the failure to identify all trigger sites or inadequate surgery on the trigger sites. In this review, we aim to provide an overview of current surgical interventions for MH at different trigger sites, including recent updates, success and failure rates, and potential causes of failure.

## Introduction and background

Migraine headache (MH) is a prevalent neurovascular disorder that affects approximately 14%-15% of the global population [1-2]. The prevalence rate is higher in women (18%) compared to men (6%), and it commonly affects individuals aged between 20 and 50 years [3-4]. Symptoms of migraine include throbbing pain in the head and face, nausea, vomiting, sensitivity to light (photophobia), and sensitivity to sound (phonophobia), with or without aura. Around 5% of MHs are considered *refractory* [5]. Chronic MH is defined by the International Headache Society Classification of Headache Disorders (ICHD-3) as a headache occurring on ≥15 days/month for more than three months, with migraine features present on ≥8 days/month [6]. The majority of these cases are treated using a combination of nonpharmacologic behavioral and pharmacologic therapies [7]. Pharmacologic treatments typically involve acute and preventive treatments which comprise non-steroidal anti-inflammatory drugs or a combination of acetaminophen, acetylsalicylic acid, and migraine-specific medications [8]. Other commonly used medications include sumatriptan and ketorolac [8]. Unfortunately, medications are not always effective, which can make managing the condition challenging. A preventive migraine drug is considered successful if it reduces the frequency of migraine attacks by at least 50% within three months [9]. Hence, it has been estimated that approximately 50% of the population experiences a significant reduction in migraine frequency [9]. In cases where pharmacologic and behavioral therapies fail to improve MH, innovative surgical interventions may be considered as an alternative option. Before 2000, there were no successful surgical techniques specifically used to deactivate MH, and understanding the pathogenesis of the condition was crucial for exploring potential theories. Although the exact cause of MH has not been clearly identified, identifying and eliminating risk factors was proven to be important in managing the disease. 

## Review

Pathogenesis of MH 

Peripheral and central mechanical theories explaining the underlying cause of MH have been extensively discussed in the literature. The central mechanism plays a crucial role in the development of MH, as it involves abnormal interactions between vasoactive neurotransmitters of trigeminal nerve (TN) terminals and dilated blood vessels. Substance P, a vasodilating peptide, is released from TN endings in response to nervous stimulation and is involved in transmitting painful stimuli in the periphery. Serotonin, another vasoactive molecule implicated in migraine pathogenesis, coexists with substance P in certain terminals of the central nervous system and is present in the trigeminal ganglia [10]. Evidence suggests that cortical and brainstem hyperexcitability, as well as cortical spreading depression, contribute to the aura preceding a headache [10-11]. Despite efforts to target the central mechanical cause of migraine through various pharmacological treatments, these approaches have yielded unsuccessful clinical results. The theories based on the central mechanism alone have not been sufficient to address the anatomical cause of MH. Therefore, the peripheral mechanism has been proposed as another potential mechanical cause of MH. Analysis of postsurgical samples from decompressed nerves in cadavers with MH revealed pathological correlations supporting this theory [12]. Electron microscopy analysis of zygomaticotemporal nerve (ZTN) segments in migraine patients showed disrupted myelin sheaths and axons, as well as poorly conveyed neurofilaments, indicating axonal pathology [12]. Unfortunately, axonal pathology is typically irreversible and challenging to treat with pharmacological interventions.

History of surgical treatment of MH based on peripheral mechanical causes 

Nowadays, the most common approach for treating MH involves a combination of avoiding migraine triggers and using prophylactic pharmacologic interventions and acute abortive and analgesic therapies [3]. While medical treatment is typically the first line of treatment for controlled cases, surgical treatment is slowly gaining popularity for refractory cases or cases that do not respond to medical treatment over a prolonged period of time. Clinical trials have been conducted to investigate the correlation between the removal of the corrugator muscle and pain improvement, which has led to a focus on migraine surgery involving the neurolysis of sensory branches of trigeminal and occipital nerves that supply the face and back of the head [3]. The inhibition of TN terminal branches and substance P release can potentially be achieved by chemically deactivating or surgically intervening in skeletal muscle function [13]. Two studies conducted in 2000-2001 provide relevant insights [13-14]. The first study demonstrated a clear correlation between surgical removal of the affected muscle and relief from migraine symptoms. Among the 314 patients who underwent forehead reconstruction, including corrugator resection, 39 had MH. After the procedure, approximately 80% of these patients reported improvements in their migraines, with lasting benefits for up to 50 months [13]. In the same year, the use of Botox injections into pericranial muscles showed a significant reduction in the frequency and severity of MH attacks [14]. In 2002, Guyuron et al. established a procedure involving the removal of corrugator muscles and transection of the ZT nerve, which resulted in a success rate of over 90% for the patients [15]. However, a common side effect of this procedure was occipital pain along the distribution of the greater occipital nerve (GON), likely caused by the original compression of the semispinalis capitis muscle during the procedure [16].

The surgical technique used to treat MH, targeting frontal, temporal, occipital, and nasal sites, has shown a high success rate in eliminating migraines [16]. Transient side effects reported were minimal and included temporal anesthesia, dryness and itching of the nose, and rarely sinus infection. A long-term evaluation of the surgery after six years confirmed its successful outcome, with approximately 60% improvement in symptoms [17]. In 2005, Guyuron et al. expanded the migraine surgical procedure to include young patients aged 9-16 years, resulting in successful improvement of MH after surgery [18]. Following Guyuron et al.'s study, multiple clinical studies have been conducted. Nagori et al. compiled these studies in a review published in 2019, which included a total of 1150 patients treated surgically for MH. In nine of these studies, Botulinum toxin was used as a diagnostic aid before surgery [19].

The studies conducted on MH trigger sites have shown variations. According to Guyuron et al., the most common trigger sites are frontal (I), temporal (II), rhinogenic (III), occipital (IV), auriculotemporal (V), and lesser occipital (VI) (Table 1) [15]. Some studies focused solely on addressing the frontal trigger point, while others performed ZT nerve avulsion or occipital nerve decompression [15,18,20-28]. Although surgical procedures between 2000 and 2019 were generally successful, there were cases where patients only experienced partial improvement or no improvement at all. The failure rate in some studies reached up to 20% [19]. When pooling data from 23 studies were collected by Nagori et al., it was found that 8%-77% of patients reported a complete elimination of headache after surgery, while 4%-34% had no relief [19]. In a study conducted by Urhan et al. in 2023, the long-term results of 93 patients who underwent migraine surgery from 2017 to 2021 were monitored [29]. The follow-up period was approximately 12 months, and it was found that 85% of patients experienced a 50% reduction in MHs, with 14% achieving complete elimination [29]. The remaining cases showed the failure of symptom relief. 

**Table 1:** MH trigger sites with recent surgical techniques. MH, migraine headache; TN, trigeminal nerve

Trigger site	Pain location	Surgical approach
Frontal trigger site (I)	Supraorbital area	Surgical or endoscopic removal of glabellar muscle group +/- SO foraminotomy with small vessel removal
Temporal trigger site (II)	Temporal area	Surgical decompression of the zygomaticotemporal (ZT) branch of TN through two small incisions about 1-5 cm long and 3.5 cm apart in the hair-bearing temple
Rhinogenic trigger site (III)	Nasal area	Septoplasty +/- turbinectomy
Occipital trigger site (IV)	Occipital and neck area	Surgical decompression of greater occipital nerve + partial removal of semispinalis capitis muscles
Auriculotemporal Site (V)	Temporal area	Surgical removal of superficial temporal artery
Lesser occipital site (VI)	Occipital area	Surgical ligation of occipital artery branches

Selection of the trigger site of MH for surgical intervention 

The accurate identification of the trigger site for surgery relies heavily on the patient's history and their ability to locate the site. Conducting a thorough history is crucial in determining the trigger sites. During the examination, the patient is asked to indicate the most frequent site of origin for their MH using one fingertip, which is then explored using a Doppler ultrasound. If an arterial Doppler signal is detected at that site, it is considered an active arterial trigger site. Confirming the presence of a trigger site can be done by observing the patient's response to a nerve block with a local anesthetic. In cases where the patient is not experiencing pain during the clinic visit, considering an injection of Botulinum toxin A at the suspected trigger site is recommended. It is advised to inject the toxin at the trigger site during each visit, starting with the most affected or common site. However, a drawback of this protocol is the time and number of visits required before surgery. Different studies have utilized various diagnostic aids, such as Botulinum toxin or local anesthetic agents, to identify the trigger site, resulting in a wide disparity in their use [3,15,23-24,27-28,30-33].

The pain originating from the frontal trigger site (I) is commonly felt in the supraorbital (SO) area during the late afternoon. Eyelid ptosis may occur during active MH episodes. This trigger site typically responds well to Botulinum toxin A injection, as well as pressure and hot/cold compresses. Surgical deactivation of this site can be achieved through either an endoscopic approach or an incision in the upper eyelid, particularly in patients with a prominent forehead (Table 1) [1]. To decompress the SO and supratrochlear nerves (STN), the glabellar muscle group must be removed. If the SON passes through an SO foramen, a foraminotomy procedure may also be necessary.

For the temporal trigger site (II), patients typically experience temporal pain and local tenderness [18]. This type of pain is commonly referred to as morning headache and is associated with clenching and grinding. The recommended approach for surgical decompression of the ZT branch of the TN involves making two small incisions, approximately 1 to 5 cm long and 3.5 cm apart, in the hair-bearing temple (Table 1) [15]. Patients with MH at the rhinogenic trigger site (III) often wake up in the morning due to pain (Table 1). This headache is usually linked to seasonal changes, exposure to allergens, or hormonal changes in women. If anti-congested agents provide long-term relief, a sinus computed tomography (CT) scan is recommended to rule out a deviated septum [18]. Depending on the findings from the intranasal examination, patients may undergo septoplasty and turbinectomy. 

At the occipital trigger site (IV), patients typically experience upper neck and occipital pain, as well as muscle tightness, which is exacerbated by intense exercise [18]. The best-preferred choice of surgery in these patients is the decompression of the GON and partial removal of semispinalis capitis muscle surrounding the nerve (Table 1). Other rare trigger sites include auriculotemporal (V) and lesser occipital (VI) sites. To identify the precise location of pain and the triggering point, Doppler ultrasound is necessary at these sites [34]. Relief from the trigger site can be achieved through surgical removal of the superficial temporal artery (STA). The lesser occipital nerve interacts with the terminal branches of the occipital artery. If Doppler ultrasound confirms the presence of the trigger site, the occipital artery can be ligated at its terminal sites with minimal exposure to the fascia (Table 1) [12].

Causes of failure of MH surgery 

The inability to eliminate MH can be attributed to various factors. One common reason for a partial clinical response is the failure to detect all trigger sites of MH or inadequate surgery on those sites [18]. According to Punjabi et al., 18% of patients who underwent MH surgery developed secondary MH at the same or different trigger sites [35]. This highlights the importance of identifying trigger sites before surgery. While identifying trigger sites may increase the success rate, other trigger terminal sites may also contribute to the poor outcome. The presence of anatomical overlaps and irritation of small peripheral branches can make it challenging to accurately locate the correct trigger site for deactivation [35]. Proper identification and management of all trigger points, along with successful nerve decompression, are crucial for achieving excellent results and minimizing failure rates. However, there may still be a subset of patients who do not respond to migraine surgery despite all treatment measures taken [36]. Gfrerer et al. found that some patients either show no improvement or only experience significant improvement after surgery, with a few patients falling into intermediate outcomes [36].

In 2023, two studies were published that provided updated information on surgical interventions for certain MH surgical techniques. The first study, conducted by Pietramaggiori et al., focused on conducting decompression of the auriculotemporal nerve (ATN) and arteries at the trigger site V [37]. This technique was carried out on 57 patients, with a success rate of approximately 79%. The second study, conducted by Raposio et al., involved surgical decompression of STN and supraorbital nerve (SON) at trigger site I in 98 patients with chronic MH [38]. The success rate for this procedure was approximately 87%.

## Conclusions

The surgical technique for decompressing the nerve through the removal of muscles or vessels as a treatment for MH has been developed based on long-term observation of patient symptoms and outcomes. While recent surgical interventions have shown high success rates in alleviating symptoms, there is still a failure rate of approximately 20%-35%. This failure rate is likely attributed to variations in surgical experience or the inability to accurately determine the origin of the trigger site. To better understand the exact mechanism of nerve compression in patients with migraine and why a subset of patients does not respond to even surgical treatment, further randomized clinical investigations and anatomical studies from various centers worldwide are necessary.
